# Hotspots and trends in acupuncture combined with non-invasive neuromodulation technology in the past 20 years: a bibliometric analysis

**DOI:** 10.3389/fneur.2025.1511655

**Published:** 2025-09-09

**Authors:** Song Li, Anhong Dai, Yihao Zhou, Xu Chen, Yizhou Chen, Li Zhou, Xiaolin Yang, Mengqi Yue, Jing Shi, Yong Qiu

**Affiliations:** ^1^Second Clinical Medical College, Heilongjiang University of Chinese Medicine, Harbin, China; ^2^Department of Acupuncture and Moxibustion, The First Affiliated Hospital of Yunnan University of Chinese Medicine, Yunnan Provincial Hospital of Traditional Chinese Medicine, Kunming, China; ^3^Department of Rehabilitation Medicine, Yan’an Hospital Affiliated to Kunming Medical University, Kunming, China; ^4^Yunnan University of Traditional Chinese Medicine, Kunming, China

**Keywords:** acupuncture, non-invasive neuromodulation technology, transcutaneous electrical acupoint stimulation, analgesia, visualization analysis, bibliometric

## Abstract

**Background:**

Non-invasive neuromodulation (NIN) technology, a promising approach in the field of neuromodulation, has been employed to address a diverse array of disorders, with an increasing volume of research emerging. Traditional Chinese acupuncture has also been utilized as a complementary and preventive therapy for various ailments. Nevertheless, the publication trends and research hotspots at the intersection of acupuncture and NIN technology remain largely unexplored. This study aims to systematically analyze the publication trends and research hotspots related to the convergence of acupuncture and NIN over the past two decades using bibliometric methods.

**Methods:**

We collected literature data on the combination of acupuncture and NIN technology from January 1, 2004, to December 31, 2023, utilizing the Web of Science Core Collection (WOSCC) database. This data was imported into CiteSpace (version 6.2. R4) and RStudio to facilitate a visual analysis of research hotspots and trends, categorized by author, country/region, affiliation, annual publication, keywords, and journal.

**Results:**

A total of 803 articles were retrieved, encompassing contributions from 55 countries, 468 research institutions, and 360 academic journals. The People’s Republic of China leads in publication volume with 390 articles, followed by Capital Medical University and Peking University, each contributing 27 articles. Among the authors, HAN JS has the highest number of co-citations, totaling 142. Additionally, the journal Evidence-Based Complementary and Alternative Medicine is noted for publishing the most papers in this field. Recent research hotspots regarding acupuncture combined with NIN technology primarily focus on topics such as “postoperative gastrointestinal function,” “postoperative analgesia,” “postoperative nausea and vomiting,” “acupuncture analgesia,” “reproductive medicine,” “inflammation,” “chronic low back pain,” and “postoperative pain.” The predominant intervention method identified is TEAS technology, which integrates acupuncture with modern electrical stimulation as an innovative clinical treatment approach.

**Conclusion:**

The publications on acupuncture combined with NIN technology have made significant progress; however, there is still a need to strengthen international academic exchanges and cooperation among scientific researchers and institutions to promote interdisciplinary collaboration and academic innovation. Furthermore, future studies should focus on enhancing the overall quality of research outcomes in this field and reinforcing research programs.

## 1 Introduction

Non-invasive neuromodulation (NIN) is defined as a technique that stimulates nerves by delivering electrical stimulation through the skin. In recent years, advancements in neuromodulation technology have significantly contributed to the advancement of brain science and have become essential tools for the diagnosis and treatment of neurological disorders, including central nervous system diseases such as stroke, Parkinson’s disease, and various mental disorders.

Neuromodulation technology employs physical modalities, including light, magnetism, electricity, and ultrasound, to modify signaling within the central nervous system, thereby influencing the excitability of neurons and associated neural networks (1). Non-invasive neuromodulation techniques (NIN), such as transcranial direct current stimulation (tDCS) (2), transcranial alternating current stimulation (tACS) (3), transcranial magnetic stimulation (TMS) (4), transcutaneous acupoint stimulation (TAS) (5), and transcranial ultrasound stimulation (TUS), have demonstrated the potential to modulate the excitability of pertinent functional areas and facilitate the reorganization of neural circuits in the brain. These techniques offer several advantages, including safety, adjustable parameters, targeted neurostimulation, and ease of operation (6–8). They have been employed to alleviate conditions such as fibromyalgia, headaches, neuromusculoskeletal pain, degenerative joint pain, chronic low back pain, depression, insomnia, and postoperative complications, including nausea, vomiting, and gastrointestinal dysfunction (9–12). In addition, NIN has a favorable preventive effect on serious complications from medication taken to reduce the significant negative effects of these medications (13). Although these techniques have a very significant placebo effect, this does not detract from the efficacy of these techniques in avoiding the serious side effects of medication, such as peptic ulcers, renal impairment, constipation, addictions, and premature deaths that result from the widespread use of NSAIDs, opioids, and benzodiazepines.

Acupuncture is a traditional healing technique that has been extensively utilized for both the prevention and treatment of a variety of clinical conditions, including stroke (14), facial paralysis (15), low back pain (16), and migraines (17). Its clinical applications primarily focus on promoting nerve recovery and providing analgesic effects. In terms of promoting nerve recovery, axons serve as the projections of neurons that conduct nerve impulses, functioning as signal transmission channels within the nervous system. Successful axonal regeneration is essential for the recovery of neurological function. Research has demonstrated that acupuncture can enhance the nutritional conditions necessary for neuron survival and axonal growth by regulating the expression of growth factors (18). Additionally, acupuncture can inhibit astrocyte scar formation, decrease the expression of myelin growth inhibitory factors such as Nogo protein and OMgp, and modulate axon guidance molecules like Sema3A and the Wnt family, thereby creating a favorable local microenvironment for axon regeneration (19, 20). Furthermore, it can regulate the PTEN/mTOR, Rho/ROCK, MAPK/ERK, and cAMP/PKA signaling pathways to stimulate the intrinsic growth capacity of axons, ultimately promoting the recovery of neurological function (21–24). In terms of analgesia, Endogenous opioid peptides (EOPs) play a significant role in pain perception, comprising at least three families: enkephalins, endorphins, and dynorphins. Acupuncture has been shown to modulate opioid levels, enhancing the concentrations of enkephalins, endorphins, and dynorphins in the cerebrospinal fluid (CSF) to achieve analgesic effects. This concept is supported by pharmacological studies and cross-tolerance research utilizing type-specific opioid blockers (25, 26). Furthermore, research indicates that acupuncture can also regulate the levels of 5-hydroxytryptamine in the spinal cord, as well as aspartic acid and glutamic acid in the dorsal horn, along with plasma substance P (SP) and calcitonin gene-related peptide (CGRP). Additionally, it can lead to an abnormal increase in arterial blood flow velocity, contributing to analgesia (27). Similarly, non-invasive neuromodulation technologies are being investigated for their potential to promote nerve recovery and enhance analgesic effects. Consequently, it is imperative to identify research hotspots and trends at the intersection of these two fields.

Bibliometric analysis serves as a crucial instrument for the comprehensive assessment and quantification of research advancements within specific scientific domains. It facilitates the mathematical and statistical evaluation of the interrelationships and impact of scholarly publications. RStudio and CiteSpace are prominent analytical tools that, when combined with knowledge mapping, are extensively utilized in the fields of information science, education, and medicine. Recent literature has reported bibliometric studies focusing on acupuncture treatments and NIN techniques (28, 29). However, there remains a notable absence of bibliometric analyses exploring the intersection of acupuncture and NIN techniques. This study employs RStudio and CiteSpace to construct a research map of published literature from the past two decades, aiming to identify frontiers and potential research hotspots at the convergence of acupuncture and NIN technology.

### 1.1 Data search

This study conducted a comprehensive search of the Web of Science Core Collection (WOSCC) from January 1, 2004, to December 31, 2023, utilizing the following MeSH terms: “acupuncture,” “repetitive transcranial magnetic stimulation,” “transcranial electric stimulation,” “transcranial alternating current stimulation,” “transcranial ultrasound stimulation,” and “transcranial direct current stimulation.” Detailed search terms and strategies employed for the WOSCC database are provided in Appendix 1. No language restrictions were applied to the studies retrieved. The results were exported as text files for subsequent visualization and analysis.

### 1.2 Inclusion/exclusion criteria

**Figure 1:**
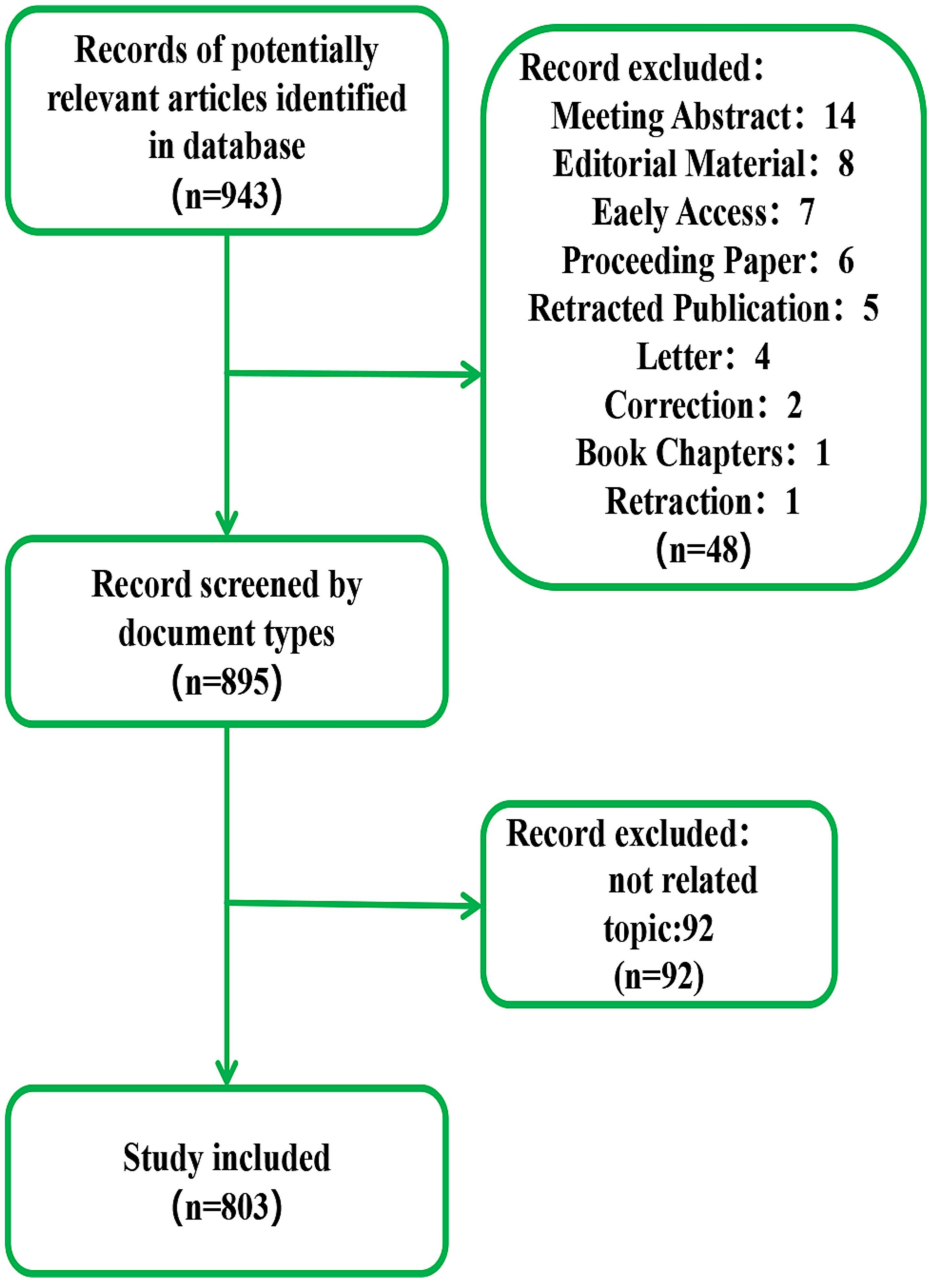
Flow chart detailing the process of article selection.

We retrieved a total of 943 articles using the developed search strategy. The inclusion criteria for these articles were as follows: (1) original research literature; (2) a publication timeframe from 2004 to 2023; (3) studies involving acupuncture and non-invasive neuromodulation techniques, including repetitive transcranial magnetic stimulation, transcranial electric stimulation, transcranial alternating current stimulation, transcranial ultrasound stimulation, and transcranial direct current stimulation; and (4) both articles and reviews. The exclusion criteria included: (1) meeting abstracts, editorial materials, early access publications, proceeding papers, retracted publications, letters, corrections, and book chapters; (2) articles focusing solely on either acupuncture or non-invasive neuromodulation; and (3) research that does not pertain to public health or medicine. Based on these criteria, we initially screened 895 papers. Two independent researchers reviewed each title, abstract, and keyword, resulting in the exclusion of 92 papers that did not align with the research content. Ultimately, 803 records were included for visual analysis (Figure 1).

### 1.3 Data analysis

This study used the bibliographic data package (version 4.1.2) of the R (version 4.2.3) and RStudio (version 2023.03.0) platforms as well as the CiteSpace (version 6.2. R4) software to visualize and analyze of publications. The analysis focuses on various factors, including publication year, country/region, journal, research institution, author, keywords, and references.

The figure illustrates various analysis objects represented by different nodes, with the diameter of each node indicating its frequency of occurrence. The lines connecting the nodes signify links, demonstrating that both nodes co-occur in the same study. Additionally, the color of the lines denotes the initial instance of their co-occurrence, while thicker lines indicate more significant connections between the two (30).

The parameters for the CiteSpace software analysis are defined as follows: (1) The analysis will cover the period from January 1, 2004, to December 31, 2023; (2) The thresholds for both ‘Top N% of each slice’ are set at 50%, with a slice duration of 1 year; (3) Clustering labels will be extracted using the log-likelihood ratio (LLR) algorithm.

## 2 Results

### 2.1 Published year

**Figure 2:**
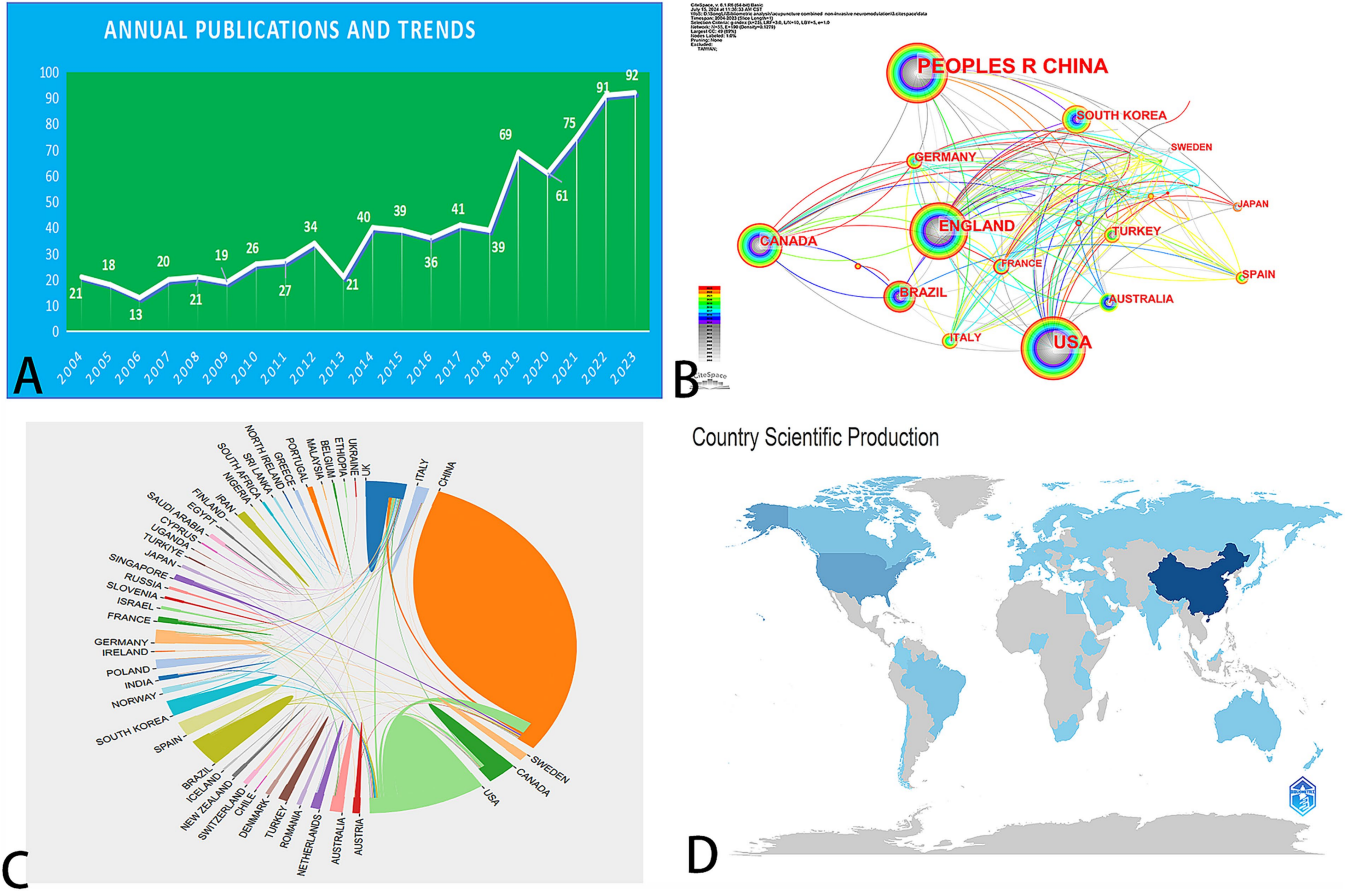
**(A)** Annual number of WoS publications in the field of acupuncture combined with non-invasive neuromodulation technology in the past 20 years. **(B–D)** National distribution of publications involving acupuncture combined with non-invasive neuromodulation technology in the past 20 years.

This study included 803 publications related to acupuncture and NIN. The number of papers published each year is illustrated in Figure 2A, which shows a range from 21 publications in 2004 to 91 in 2023. Overall, there is a discernible upward trend in publications concerning acupuncture and NIN, although minor fluctuations were observed in 2006, 2013, and 2020. Notably, 2006 marked the year with the fewest publications in the past two decades, with only 13 papers, while 2022 saw the highest output, with 91 publications—seven times the number published in 2006.

### 2.2 Countries/regional

CiteSpace and RStudio were utilized to conduct a visual analysis of the countries and regions contributing to publications on acupuncture combined with NIN techniques over the past two decades (Figures 2B–D). The People’s Republic of China led the publication count with 390 papers, representing 48.57% of the total output. This was followed by the United States (21.54%), England (9.09%), Canada (5.23%), and Brazil (4.36%) (Table 1). In terms of international collaboration, England exhibited the highest contact centrality (0.6), followed by the United States (0.23), the People’s Republic of China (0.13), and Brazil (0.03). The link centrality of other countries was relatively low, indicating a notable level of cooperation between England and its counterparts. The visualized international collaboration network illustrates that England has established significant cooperative ties with both the United States and the People’s Republic of China. These findings underscore the pivotal role of these three nations in this research domain; however, there remains a need for enhanced international collaboration among countries.

**Table 1:** The 5 countries/regions that publish the most papers involving acupuncture combined with non-invasive neuromodulation technology.

Ranking	Countries/Regional	Counts	Centrality	%(of 803)
1	Peoples Republic of China	390	0.13	48.57%
2	United States	173	0.23	21.54%
3	England	73	0.60	9.09%
4	Canada	42	0.11	5.23%
5	Brazil	35	0.03	4.36%

### 2.3 Journals analysis

**Figure 3:**
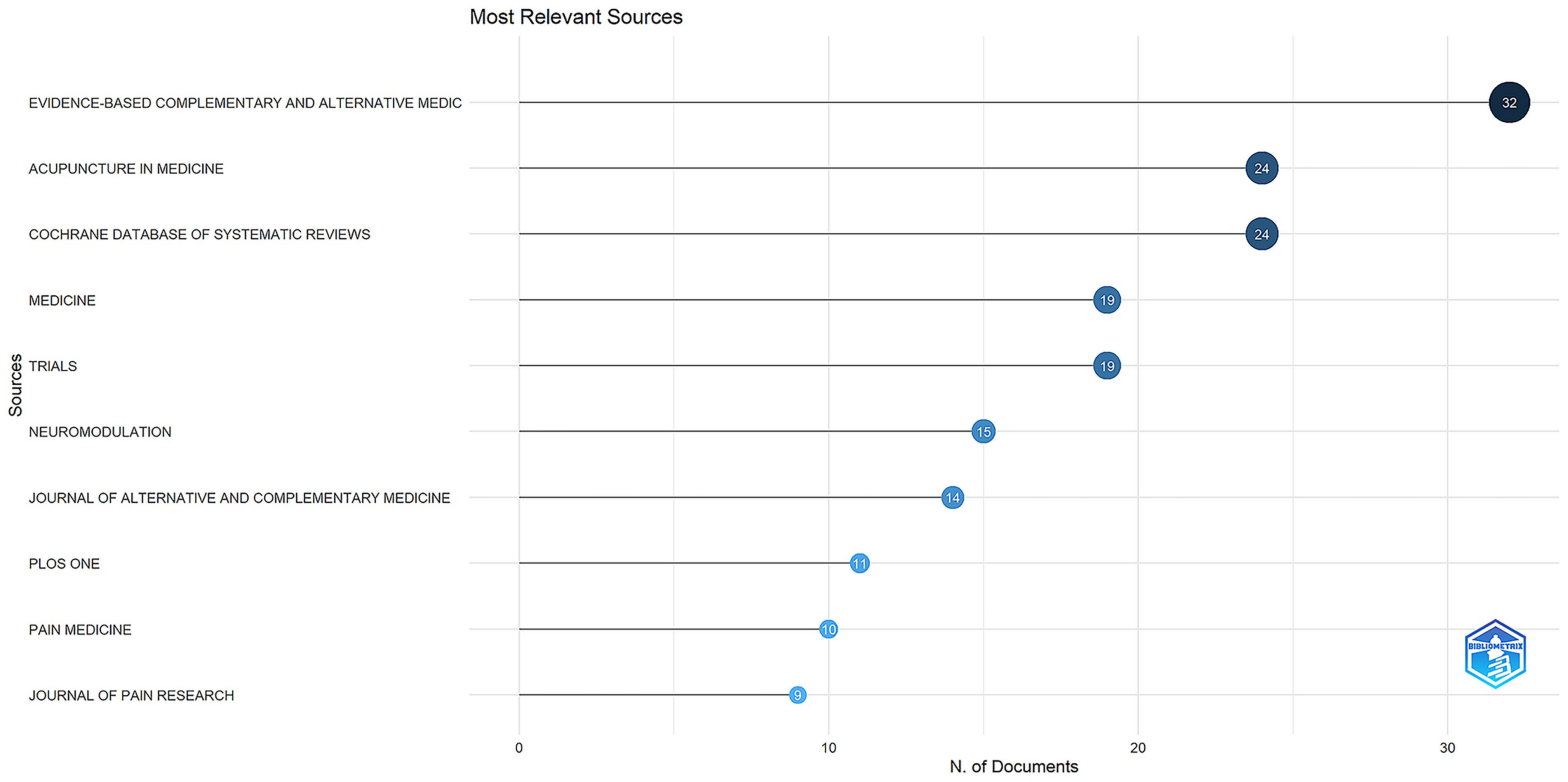
The 10 journals with the highest frequency of publications on acupuncture combined with non-invasive neuromodulation technology in the past 20 years.

We conducted a comprehensive analysis of the relevant literature, ultimately including 803 articles across 360 journals from various countries. The top 10 most productive journals are detailed in Table 2 and illustrated in Figure 3. The journal with the highest publication count is “Evidence-Based Complementary and Alternative Medicine,” which published 32 articles (3.99%), followed by “Acupuncture in Medicine” and “Cochrane Database of Systematic Reviews,” each with 24 articles (2.99%). “Medicine” and “Trials” both published 19 articles (2.37%). This indicates the significant role of “Evidence-Based Complementary and Alternative Medicine” in the field despite its exclusion from the Science Citation Index (SCI) in 2023. The impact factors of the top 10 journals range from 1.3 to 8.8, with their Journal Citation Reports (JCR) categories distributed as follows: Q1 (5.60%), Q2 (8.34%), and Q3 (4.11%). These findings suggest that future research may yield a higher volume of publications, underscoring the ongoing need for high-quality research.

**Table 2:** The 10 journals with the highest frequency of publications on acupuncture combined with non-invasive neuromodulation technology.

Ranking	Journal	Articles	IF(2023) (JCR)	% (of 803)
1	Evidence-Based Complementary And Alternative Medicine	32	0	3.99%
2	Acupuncture In Medicine	24	2.4 (Q2)	2.99%
3	Cochrane Database of Systematic Reviews	24	8.8 (Q1)	2.99%
4	Medicine	19	1.3 (Q2)	2.37%
5	Trials	19	2.0 (Q3)	2.37%
6	Neuromodulation	15	3.2 (Q2)	1.88%
7	Journal of Alternative and Complementary Medicine	14	1.3 (Q3)	1.74%
8	Plos One	11	2.9 (Q1)	1.37%
9	Pain Medicine	10	2.9 (Q1)	1.25%
10	Journal of Pain Research	9	2.5 (Q2)	1.12%

### 2.4 Co-cited journals

**Figure 4:**
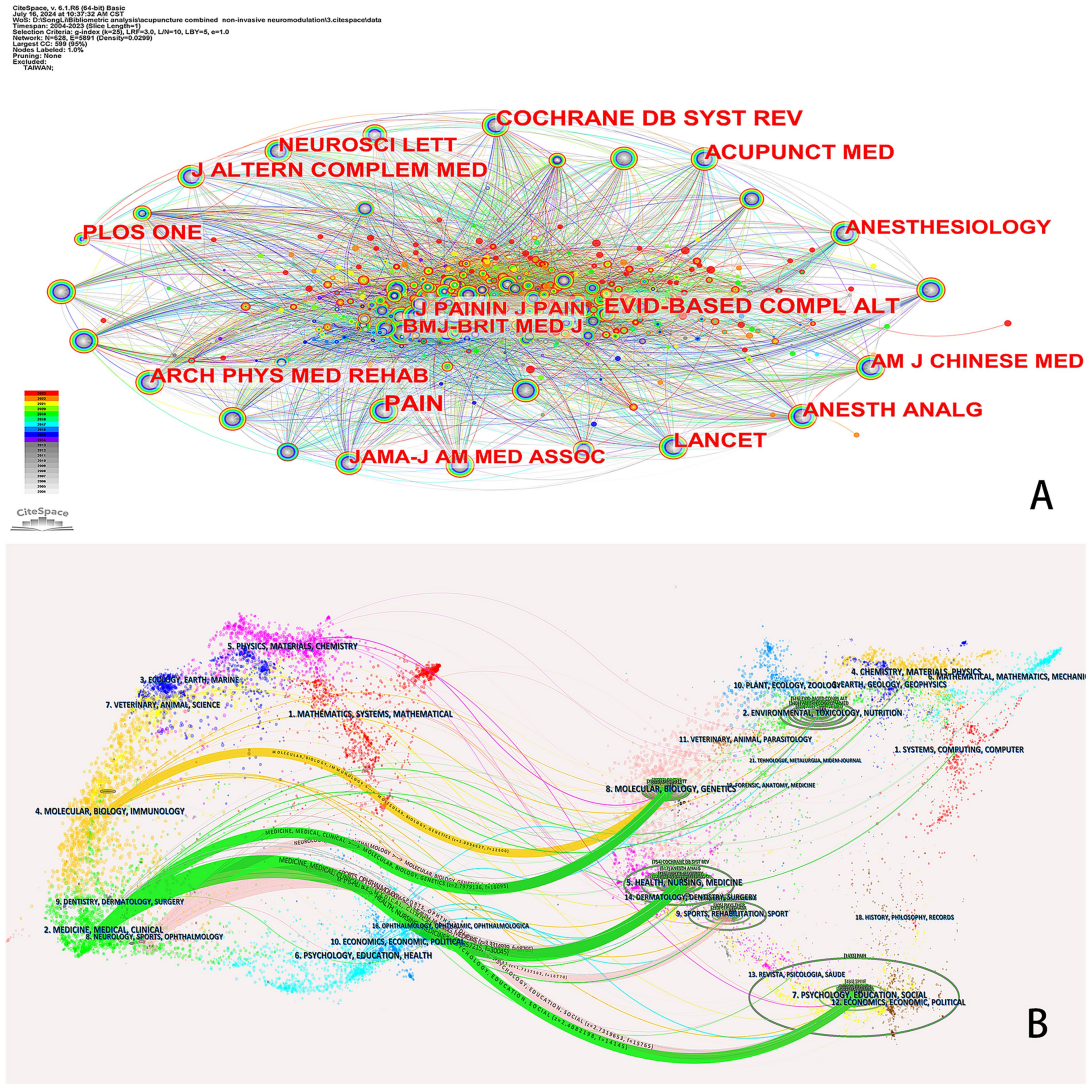
**(A)** Distribution of co-cited journals in acupuncture combined with non-invasive neuromodulation technology. **(B)** The dual-map overlay of journals related to PE research.

Table 3 presents the top 10 journals ranked by the frequency of neighborhood co-citation related to acupuncture combined with NIN technology. The journal PAIN emerges as the most frequently cited, with a total of 358 citations, followed by the Cochrane Database of Systematic Reviews with 278 citations and Evidence-based Complementary and Alternative Medicine with 277 citations. In contrast, the citation frequencies of the remaining journals are all below 250 citations. This finding underscores the significant influence of these three journals within the domain of acupuncture integrated with NIN technology. Figure 4A illustrates the network diagram of co-citing journals, while Figure 4B displays a dual map overlay of a journal, with the left and right sides representing the citation map and the cited journal map, respectively. The tags on the map denote the disciplines encompassed by the journal, and the lines connecting the left to the right signify citation links. There are 4 main paths, Path 1: MOLECULAR/BIOLOGY/IMMUNOLOGY cites the field of MOLECULAR/BIOLOGY/GENETICS (*Z* = 2.0994627, *f* = 12,600); Path 2: MEDICINE/MEDICAL/CLINICAL cites the field of MOLECULAR/BIOLOGY/GENETICS (*Z* = 2.7979136, *f* = 16,095). Path 3: MEDICINE/MEDICAL/CLINICAL cites the field of HEALTH/NURSING/MEDICINE (*Z* = 5.5857215, *f* = 30,045); Path 4: MEDICINE/MEDICAL/CLINICAL cites the field of PSYCHOLOGY/EDUCATION/SOCIAL (*Z* = 2.4082198, *f* = 14,145).

**Table 3:** The 10 most frequently cited journals on acupuncture combined with non-invasive neuromodulation technology.

Ranking	Journal	Centrality	Counts
1	Pain	0.02	358
2	Cochrane DB Syst Rev	0.04	278
3	Evid-Based Compl Alt	0.03	277
4	Acupunct Med	0.04	231
5	J Altern Complem Med	0.03	222
6	PLoS One	0.01	214
7	Lancet	0.03	213
8	Anesth Analg	0.02	200
9	Arch Phys Med Rehab	0.04	193
10	Clin J Pain	0.03	180

### 2.5 Author’s and co-authorship analysis

**Figure 5:**
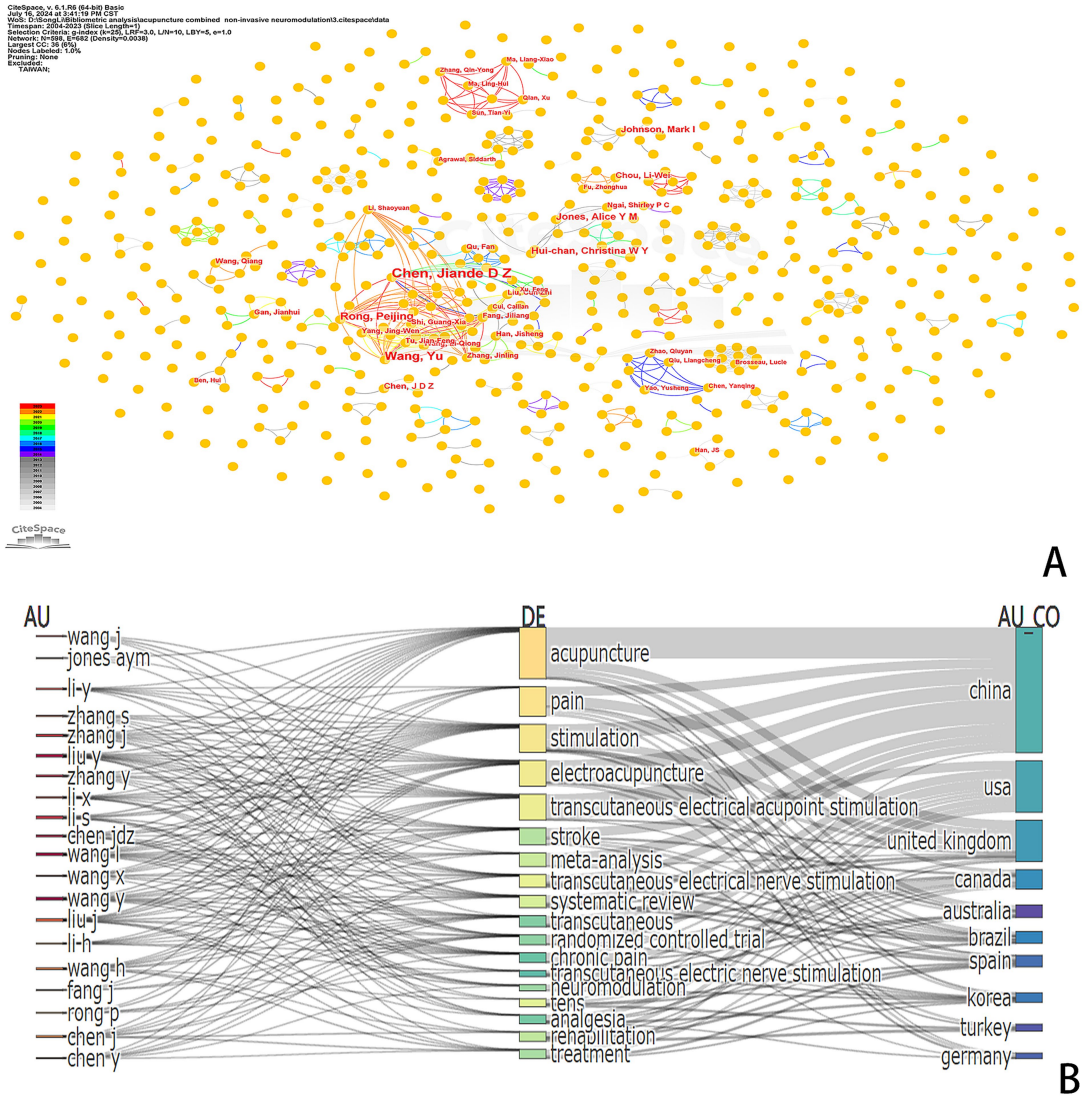
**(A)** Author distribution in the research field of acupuncture combined with non-invasive neuromodulation. **(B)** Analysis of the relationship between authors, keywords, and countries.

We utilized RStudio and CiteSpace to perform a visual analysis of the 803 publications included in our study, which involved a total of 598 authors. The author visualization illustrates the extent of collaboration among authors, offering insights into influential research groups and potential collaborators (Figure 5A). In terms of publication output, Chen, Jiangde D Z, and their team are the leading authors in this field, having published a total of 20 papers. They are followed by Wang and Yu, who have published 13 articles, while other authors have published fewer than 10 articles each. Table 4 presents the 10 most productive and cited authors in the field. Among the most cited authors, excluding those with unknown citation counts, we observe that HAN JS ranks first with 142 citations, followed by MELZACK R with 90 citations, and SLUKA KA with 63 citations. The citation frequency for the remaining authors is below 50 citations.

**Table 4:** Top 10 authors and co-cited authors of the study of acupuncture combined with non-invasive neuromodulation technology.

Ranking	Authors	Count	Ranking	Frequency	Co-cited author
1	Chen, Jiande D Z	20	1	142	Han JS
2	Wang, Yu	13	2	90	Melzack R
3	Rong, Peijing	9	3	63	Sluka KA
4	Hui-chan, Christina W Y	7	4	47	Wang H
5	Jones, Alice Y M	7	5	44	Johnson MI
6	Johnson, Mark I	6	6	36	Bjordal JM
7	Chen, J D Z	5	7	34	Chen L
8	Chou, Li-Wei	5	8	32	Moher D
9	Ngai, Shirley P C	4	9	31	Hamza MA
10	Han, Jisheng	4	10	31	Higgins JPT

We utilized RStudio to create a Three-Field Plot (TFP) that illustrates the relationships among authors, keywords, and countries (Figure 5B). The area of each rectangle corresponds to the number of publications, while the connecting lines represent the correlation between academic strengths; a greater number of links indicates more extensive research activity. Notably, Chen, Jiangde D Z exhibits the most comprehensive research scope among authors in this field, demonstrating significant associations with keywords such as acupuncture, stimulation, electroacupuncture, stroke, transcutaneous, chronic pain, and neuromodulation.

In the realm of country analysis, Chinese authors have made invaluable contributions through numerous publications that examine various facets of the field. While the volume of articles published by countries such as the United States is comparatively limited, the primary research areas encompass “electroacupuncture,” “acupuncture,” “transcutaneous stimulation,” “chronic pain,” “pain management,” “neuromodulation,” and other related domains.

### 2.6 Institutional analysis

**Figure 6:**
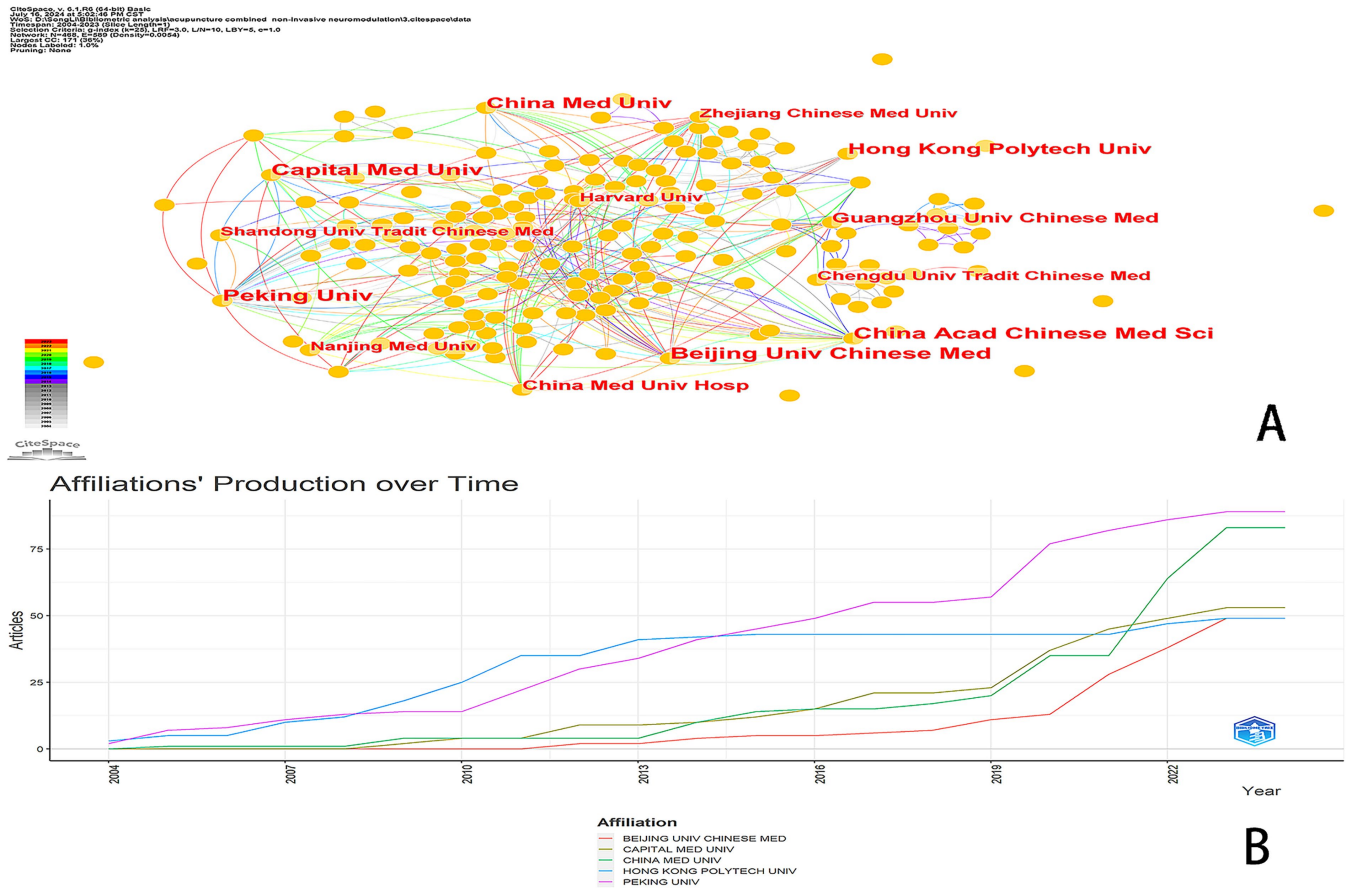
Affiliation analysis. **(A)** Network diagram of publishing institutions using CiteSpace. **(B)** Affiliation production over time depicted using bibliometrics.

A total of 468 institutions participated in research concerning the integration of acupuncture and NIN technology (Figure 6A). While collaboration among these agencies is robust, there remains room for improvement. Figure 6B illustrates the five affiliated institutions that have recently been active in publishing scholarly articles. Table 5 enumerates the five most prolific institutions in this field. Capital Medical University and Peking University lead with the highest publication counts, contributing 3.36% and 27 articles, respectively. They are closely followed by Beijing University Chinese University with 3.28% (26 articles), China Academy of Chinese Medical Sciences with 3.11% (25 articles), and Hong Kong Polytechnic University along with China Medical University, each contributing 2.74% (22 articles). Guangzhou University of Chinese Medicine reports 1.88% with 15 articles. Notably, Peking University exhibits the highest centrality score of 0.27, indicating that it engages in collaborations most frequently with other institutions, positioning it as a potential partner for future research initiatives.

**Table 5:** Top 5 institutions publishing the most papers in the field of acupuncture combined with non-invasive neuromodulation technology.

Ranking	Institution	Centrality	Counts	% (of 803)
1	Capital Med Univ	0.03	27	3.36%
1	Peking Univ	0.10	27	3.36%
2	Beijing Univ Chinese Med	0.04	26	3.28%
3	China Acad Chinese Med Sci	0.06	25	3.11%
4	Hong Kong Polytech Univ	0.02	22	2.74%
4	China Med Univ	0.03	22	2.74%
5	Guangzhou Univ Chinese Med	0.03	15	1.88%

### 2.7 Keyword analysis

**Figure 7:**
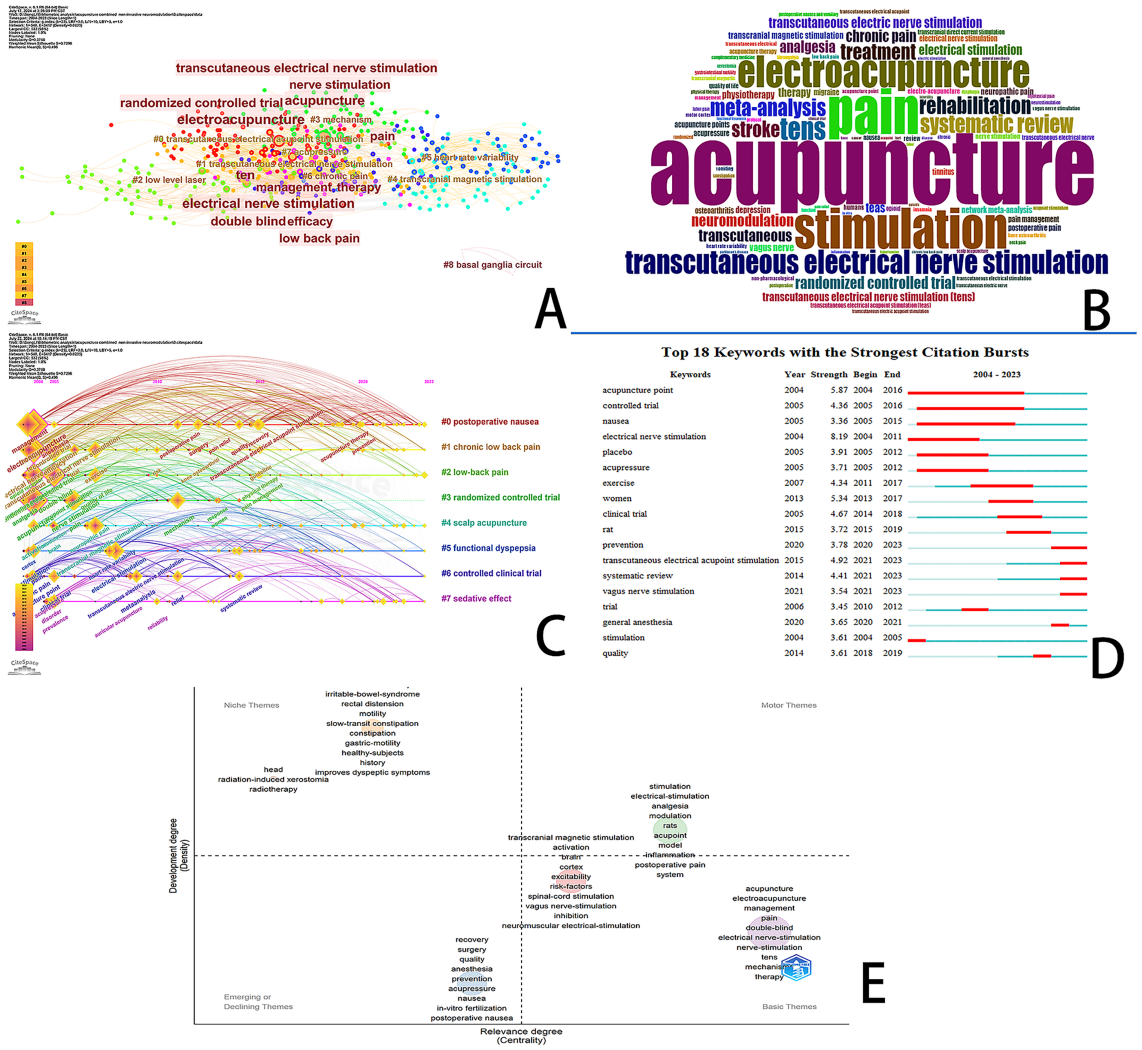
Keyword analysis **(A)** Co-occurring keyword clustering diagram. **(B)** Keyword cloud of co-occurring keywords. **(C)** Timeline of keywords. **(D)** The top 18 keywords with the strongest citation burst. **(E)** Thematic tendency map.

To comprehend the core content and prominent topics in this field, we perform an analysis of keywords. Table 6 presents the top 10 most frequently occurring and essential author keywords. The primary research keywords identified include acupuncture (294), electroacupuncture (199), management (91), pain (80), transcranial magnetic stimulation (79), electrical nerve stimulation (78), double-blind (73), nerve stimulation (66), randomized controlled trial (65), and mechanism (60). Consequently, we anticipate that future research hotspots in the domain of acupuncture combined with NIN technology will concentrate on themes such as “electroacupuncture,” “management,” “pain,” “mechanism,” and “electrical nerve stimulation.” Generally, clusters with silhouette values greater than 0.5 are considered reasonable, while values exceeding 0.7 indicate convincing clustering. The weighted average silhouette for this cluster is S = 0.7296, suggesting that the clustering is indeed convincing. We employed CiteSpace and RStudio software to conduct a keyword co-occurrence analysis based on the authors’ keywords (Figures 7A–C). A total of 540 keywords within this field were clustered and analyzed, with the first eight clusters visualized alongside the evolution timeline of the keywords (Figures 7A,C). The eight main clusters identified are #0 postoperative nausea, #1 chronic low back pain, #2 low back pain, #3 randomized controlled trial, #4 scalp acupuncture, #5 functional dyspepsia, #6 controlled clinical trial, and #7 sedative effect.

**Table 6:** Top 10 co-occurring keywords in the research field of acupuncture combined with non-invasive neuromodulation technology.

Ranking	Keyword	Counts	Centrality	Ranking	Keyword	Counts	Centrality
1	Acupuncture	294	0.17	1	Electroacupuncture	199	0.24
2	Electroacupuncture	199	0.24	2	Acupuncture	294	0.17
3	Management	91	0.06	3	Electrical nerve stimulation	78	0.15
4	Pain	80	0.07	4	Randomized controlled trial	65	0.15
5	Transcranial magnetic stimulation	79	0.09	5	Double blind	73	0.11
6	Electrical nerve stimulation	78	0.15	6	Transcranial magnetic stimulation	79	0.09
7	Double blind	73	0.11	7	Nerve stimulation	66	0.08
8	Nerve stimulation	66	0.08	8	Transcutaneous electrical nerve stimulation	54	0.08
9	Randomized controlled trial	65	0.15	9	Electrical stimulation	52	0.08
10	Mechanism	60	0.07	10	Mechanism	60	0.07

“Burst words” refer to keywords that experience heightened frequency of use during specific time periods, thereby emerging as significant topics of research. For instance, Figure 7D illustrates the top 18 keywords exhibiting the most substantial citation explosions. Notably, terms such as “acupuncture point,” “controlled trial,” “nausea,” “electrical nerve stimulation,” “acupressure,” “exercise,” “general anesthesia,” “clinical trial,” “prevention,” “vagus nerve stimulation,” “systematic review,” and “transcutaneous electrical acupoint stimulation” demonstrate elevated burst intensity. Among these, “transcutaneous electrical acupoint stimulation,” “prevention,” and “vagus nerve stimulation” are currently recognized as research hotspots and are expected to remain relevant in the foreseeable future, thereby offering valuable research directions for emerging scholars in this domain.

We utilized RStudio to analyze the “Trends of Themes” in the field of acupuncture in conjunction with NIN technology (Figure 7E). The results indicate that contemporary topics, including “stimulation,” “electrical stimulation,” “analgesia,” “modulation,” “rats,” “acupoint,” “model,” “inflammation,” “postoperative pain,” and “system,” are rapidly developing and emerging themes.

### 2.8 Reference co-citation analysis

Over the past two decades, a total of 797 documents related to the integration of acupuncture and NIN technology have been cited in the literature. Among the 10 most frequently cited references, six are articles, and four are reviews. Eight of these studies specifically examine the combination of acupuncture with NIN technology, with transcutaneous acupoint electrical stimulation identified as the primary intervention method. The majority of research focuses on the effects of this combination on postoperative gastrointestinal function (35), postoperative analgesia (36), postoperative nausea and vomiting (10), as well as perioperative anesthetic dosage, recovery, complications, and prognosis (37–40). Additionally, the literature addresses topics such as acupuncture analgesia (27)and reproductive medicine (41). These findings suggest that future research in this domain is likely to concentrate on areas such as analgesia and postoperative complications.

**Figure 8:**
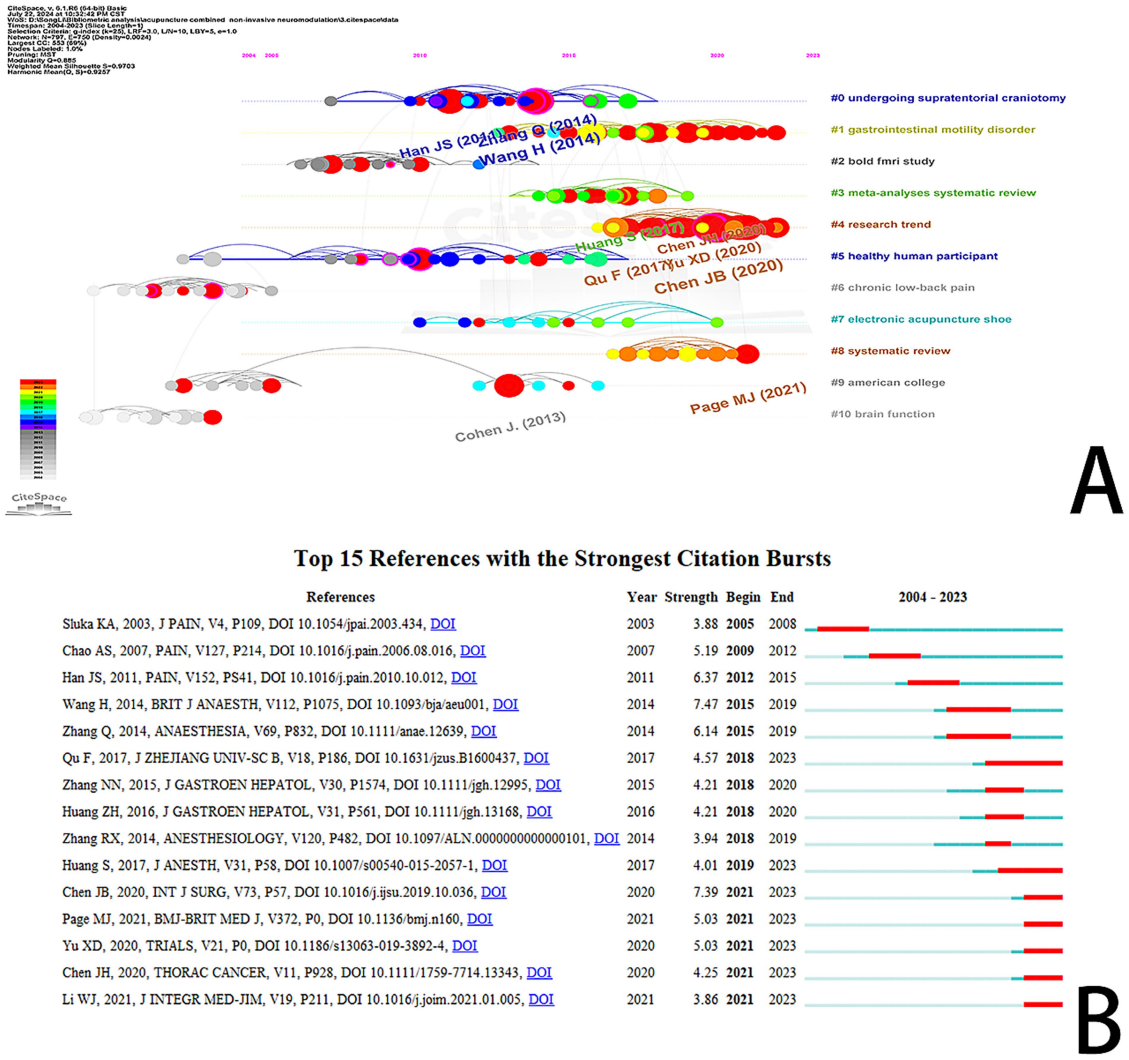
**(A)** Timeline diagram of references in research fields related to acupuncture combined with non-invasive neuromodulation technology. **(B)** Top 15 references with the strongest citation burst.

We conducted a co-citation analysis of the references, presenting the findings in the form of a timeline. Figure 8A illustrates the 11 major groups identified through this analysis. During the period from 1999 to 2005, the co-cited references predominantly focused on topics such as “chronic low-back pain,” “American college,” and “brain function,” which indicates the early exploration of acupuncture in conjunction with NIN technology. Subsequently, from approximately 2002 to 2018, the focus shifted to subjects including “undergoing supratentorial craniotomy,” “blood fMRI study,” and “healthy human participant.” The most recent co-citation clustering, beginning in 2010, emphasizes “gastrointestinal” and “electronic acupuncture shoe,” suggesting that these are current research hotspots. Figure 8B displays the top 15 references with the strongest citation bursts.

## 3 Discussion

### 3.1 General information

This study examined 803 papers published over the past 20 years, revealing a rapid annual increase in both the number of publications and citations. This trend underscores the strong interest among researchers in the integration of acupuncture with NIN technology, which reflects the synergy between traditional acupuncture practices and modern technological advancements in clinical treatment. Among the top 10 contributing countries and regions, the People’s Republic of China accounts for 48.57% of the published papers, with Capital Medical University leading the institution with the highest volume of literature in this domain. This prominence may be attributed to the historical roots of acupuncture therapy in ancient China, contributing to its greater recognition and acceptance within the country. As science and technology continue to evolve, there is a growing trend toward the integration of traditional and modern medicine driven by clinical needs. Consequently, an increasing number of researchers are innovating traditional acupuncture theories and techniques, investigating potential targets, mechanisms of action, and treatment methodologies for various diseases. This research aims to optimize clinical treatment plans, enhance therapeutic efficacy, and alleviate the burden of illness. Notably, Chen, Jiangde D Z, and their team led the author distribution of 20 published papers. According to Price’s Law (42), the minimum number of publications required for a core author is given by *N* = 0.749 (M max is the most prolific author’s publication), resulting in an approximation of N ≈ 3.34. An analysis of the published documents reveals that only 37 out of 598 authors have published more than three articles, which constitutes 6.19% of the total publications, and indicates that a core group of authors has yet to emerge in this research field. Currently, scholars investigating the integration of acupuncture with non-invasive neuromodulation (NIN) technology are predominantly located in Asia. It is imperative for researchers to enhance collaboration and exchange among scholars from various countries to further investigate the benefits and mechanisms of acupuncture combined with NIN technology in the treatment of prevalent diseases.

Academic research within a specific field must be anchored in established theories and robust evidence. References serve as crucial indicators of the research’s foundational basis and the supporting evidence pertinent to the discipline. The most frequently cited work in this area is the publication by Chen, Jiabao (10), and his colleagues, appearing in the International Journal of Surgery (Impact Factor = 12.5). This article, titled “Transcutaneous Electrical Acupoint Stimulation for Preventing Postoperative Nausea and Vomiting After General Anesthesia: A meta-Analysis of Randomized Controlled Trials,” reviews 14 clinical studies focused on transcutaneous electrical acupoint stimulation (TEAS) interventions designed to mitigate postoperative nausea and vomiting (PONV) in patients recovering from general anesthesia. The findings suggest that TEAS represents a viable approach that can be incorporated into multimodal management strategies aimed at preventing PONV, postoperative pain (PON), and emergence agitation (EAE). Additionally, TEAS is linked to a reduced requirement for antiemetic emergency care and a lower incidence of adverse effects following anesthesia. The second most cited article is a study conducted by Wang et al. (38), which investigated the effects of Hegu (LI4), Neiguan (PC6), and Zusanli (ST36) acupoints prior to anesthesia in patients undergoing sinus incision. The study implemented a 30-min transcutaneous electrical acupoint stimulation (TEAS) intervention at 6–9 mA and 2/10 Hz. Perioperative hemodynamics were monitored, peripheral blood samples were collected, and the levels of surgical stress mediators, intraoperative remifentanil consumption, recovery quality, and post-anesthesia-related side effects were assessed. The findings indicated that TEAS effectively reduces remifentanil consumption during surgery and alleviates postoperative side effects in patients undergoing sinus incision. However, the study lacked a blank control group, which would consist of conventional treatment without any intervention. Consequently, the differences between the TEAS stimulation group and the control group, as well as the blank control group, cannot be clearly delineated. It remains uncertain whether needle stimulation would yield similar effects. Thus, the study has certain limitations that diminish the credibility of its findings. Similar methodological issues are present in other papers selected for this review. In summary, the quality of research in this field requires further optimization and enhancement.

### 3.2 Hot research topics

We utilized CiteSpace and R Studio to visualize and analyze keywords, aiming to explore the research trends and hotspots associated with the integration of acupuncture and non-invasive neuromodulation technology. As anticipated, common keywords such as “acupuncture,” “electroacupuncture,” “treatment,” “transcranial magnetic stimulation,” and “neurostimulation” emerged prominently. Both clinical and fundamental research increasingly focus on elucidating the mechanisms of disease and the pathological changes that occur following intervention and treatment, with the objective of optimizing clinical diagnosis and therapeutic strategies. Predominant research methodologies in this domain include “controlled trials,” “double-blind clinical trials,” and “systematic reviews.” The combination of acupuncture with non-invasive neuromodulation (NIN) technology is frequently employed to achieve disease prevention through various means, including “acupoints,” “nerve electrical stimulation,” “acupoint pressure,” “exercise,” “vagus nerve stimulation,” and “transcutaneous acupoint electrical stimulation,” all of which contribute to therapeutic effects.

Through an analysis of common keywords and ‘burst word’ fields, we identified that current research in this area predominantly focuses on topics such as ‘chronic low back pain,’ ‘low back pain,’ ‘functional dyspepsia,’ ‘analgesia,’ ‘regulation,’ ‘rat models,’ ‘acupoints,’ ‘inflammation,’ and ‘postoperative pain.’ Notably, the regulatory mechanisms underlying ‘analgesia’ are complex, particularly concerning chronic pain, and are primarily associated with several key factors: (1) Neuroinflammation: an increased release of inflammatory mediators including prostaglandins, histamine, and bradykinin; (2) Neurotransmission Quality: an imbalanced release of neurotransmitters such as 5-hydroxytryptamine, norepinephrine, epinephrine, glutamate, and *γ*-aminobutyric acid; (3) Pain Sensitization: both peripheral and central sensitization processes; (4) Ion Channels: alterations in the expression of ion channels, including sodium, potassium, and calcium ions; and (5) Activation of Glial Cells: involvement of microglia and astrocytes (43–47). The primary mechanism underlying this phenomenon is central pain sensitization, which is intricately linked to alterations in synaptic plasticity. Increases in neuronal excitability, synaptic efficacy, and inhibition can all contribute to the enhanced function of nociceptive pathways. This central sensitization may result in the persistence of pain, ultimately leading to chronic and intractable pain conditions (44). Research has confirmed that NIN technology is effective in treating various types of chronic pain and holds significant practical applications and promising prospects (48). This technology primarily stimulates the fast mechanoreceptive large nerve A *β* fibers to inhibit the transmission of slower nociceptive signals conveyed by the large nerve A *δ* fibers and C fibers to higher centers of the brain, resulting in analgesia. It not only regulates peripheral pathways but also activates central pathways, modulates endogenous neurotransmitters, and influences the plasticity of NMDA receptors. Furthermore, it prolongs analgesic effects by modulating pain signals and reducing neuronal sensitization and neural remodeling (49–51). Notably, clinical trials have demonstrated that acupuncture operates through a similar mechanism for the treatment of chronic pain (52–54). The analogous analgesic mechanisms may be pivotal to the integration and advancement of acupuncture with NIN technology. An analysis of research conducted over the past 20 years reveals that studies on analgesia in this domain predominantly concentrate on three main areas: “low back pain,” “chronic low back pain,” and “postoperative pain.” Additionally, emerging topics such as “functional dyspepsia,” “regulation,” “inflammation,” “rat models,” “acupoints,” and “models” present novel avenues for future research in this field.

In terms of treatment methods, our findings indicate that Transcutaneous Electrical Acupoint Stimulation (TEAS) is the primary approach. TEAS effectively integrates the benefits of traditional Chinese acupoints with transcutaneous electrical nerve stimulation. Notably, when compared to electroacupuncture and traditional acupuncture, TEAS not only demonstrates comparable analgesic effects but also offers advantages in terms of simplicity, safety, and non-invasiveness (55–57). Furthermore, TEAS allows for more precise control of acupuncture parameters, such as frequency, than traditional acupuncture techniques. This method represents a fusion of traditional acupuncture practices and modern technology. Currently, TEAS stands out as an innovative approach that combines acupuncture with NIN technology. The research surrounding this therapy is gaining prominence and may emerge as a focal point for ongoing exploration and development within this field in the future.

In contrast to TEAS, which exerts its therapeutic effects by stimulating specific acupoints to modulate neurotransmitter release and inflammatory pathways, TMS and tDCS focus on direct modulation of the central nervous system - TMS relies on a time-varying magnetic field to induce an induced electric current in the cortex to modulate neural plasticity, whereas tDCS utilizes weak DC fields to directly modulate neuronal excitability in the cortex. TEAS is a highly effective and safe adjunctive therapy that has demonstrated significant clinical value in reducing the risk of opioid dependence and preventing perioperative neurocognitive disorders (PND). Compared to TMS and tDCS, which focus on direct central nervous system(CNS) modulation, TEAS also shows potential in the prevention and treatment of CNS disorders such as Parkinson’s disease (58), and chronic cancer pain (59). In particular, TEAS has outstanding potential for perioperative analgesia, PND prevention, and postoperative rehabilitation due to its simplicity, low cost, and ease of integration into the perioperative management process (38, 60). Therefore, we believe that current and future research will focus on exploring the role of TEAS in comprehensive perioperative management. As for TMS and tDCS technologies, the research focus is more likely to be on the long-term neuromodulation treatment of chronic central nervous system diseases such as Parkinson’s disease and chronic cancer pain.

The potential of neuromodulation technology in the treatment of diseases, especially brain disorders, has become increasingly prominent in recent years, offering new therapeutic options and broad prospects for patients. Since Grossman et al. (34) published a paper in Cell in 2017 proposing “Temporal Interference” (TI) stimulation, this innovative therapy has attracted widespread attention as an electrical stimulation technology among emerging therapeutic tools due to its ability to precisely target deeper regions of pathogenicity. Multi-polar Timing Interference (mTI) is a modified form of TI that allows for independent control of the size of the stimulation area and the intensity of the stimulation. By independently controlling the size of the stimulation area to precisely localize the lesion, the treatment can be more targeted, thus enhancing the therapeutic effect and reducing the impact on non-targeted areas (61). Currently, TI technology has shown potential application in the treatment of Parkinson’s disease, sleep apnea and other diseases (32, 62). Although this technology is still in its early stages and there are currently many further studies in computational modeling, animal studies, and human trials (32, 33, 61, 62), we still believe that research in areas related to the combination of acupuncture techniques and TI electrical stimulation may be a hotspot for future research in this field.

In analyzing the 10 most frequently cited documents in this field, the study identified transcutaneous acupoint electrical stimulation (TEAS) technology as the primary intervention method. The topics covered include #1 Postoperative gastrointestinal dysfunction (PGD), which is one of the most common complications in patients undergoing major abdominal surgery (63). Currently, the most commonly employed treatment methods are early exercise and the administration of nonsteroidal anti-inflammatory drugs (NSAIDs), alongside a reduction in opioid use (64, 65). These strategies aim to enhance the recovery of gastrointestinal function. However, the side effects associated with NSAIDs can limit their application. Brain-gut peptides are small molecules distributed throughout the gastrointestinal tract and central nervous system, playing a crucial role in regulating gastrointestinal function (66). Substance P (SP), a member of the tachykinin family, is involved in various physiological processes within the gastrointestinal system. An excessive release of SP by the body following surgery can lead to gastrointestinal dysfunction (9). Studies have confirmed that TEAS treatment can downregulate SP levels, thus facilitating the recovery of postoperative gastrointestinal function (35). #2 Endogenous opioid peptides (EOP) are believed to be closely linked to pain perception. Continuous electroacupuncture (EA) or TEAS, through electrical stimulation at specific frequencies, can promote the release of three endogenous opioid peptides (enkephalin, endorphin, and dynorphin) in cerebrospinal fluid (CSF), thereby delaying the onset of analgesic tolerance. Research indicates that the analgesic mechanism of acupuncture is associated with the activation of the endogenous opioid system (25, 26). The arcuate nucleus of the hypothalamus (ARC) is an important component of the endogenous opioid peptide system. The arcuate nucleus of the ARC is an important component of the endogenous opioid peptide system. As an integration center, the periaqueductal gray matter (PAG) of the hypothalamus is capable of receiving nerve fiber projections from the frontal cortex, insular cortex, and arcuate nucleus. Using 2 Hz electroacupuncture to stimulate the foot-sanli point (ST36), researchers found that it increased *β*-endorphin levels and proopiomelanocortin (POMC) transcript levels. Activation of the cAMP-PKA-CREB signaling pathway in POMC neurons within the ARC upregulated c-Fos and Jun B expression, thereby promoting *β*-endorphin synthesis; subsequently, the produced β-endorphins were projected through a specific neural circuit - i.e., from POMC neurons in the ARC to PAG GABA ergic neurons - is transferred to the pain integration center PAG, from neuromodulatory plasticity; meanwhile, optogenetic and chemical genetics experiments confirmed that inhibition of the excitability of the ARCPOMC→PAGGABA circuit significantly impairs the transfer of β-endorphin and the analgesic effect of EA, confirming that this circuit is the key factor in β- endorphin-mediated EA analgesia in the critical transport pathway (67). Furthermore, high-intensity EA exhibits broader and longer-lasting analgesic effects compared to low-intensity EA, likely due to the activation of A*δ* and C fibers, which stimulate the brainstem’s negative feedback system in response to noxious stimuli (36). #3 The antiemetic effects of acupuncture may be attributed to alterations in the activity of neurochemicals within the central nervous system, including endorphins, serotonin, and norepinephrine. Upon stimulation of acupuncture points, low-frequency electrical stimulation is generated, activating A-β and A-δ fibers, which leads to the release of endorphins into the central nervous system, ultimately enhancing endogenous anti-vomiting pathways. A review of the activation of central serotonin and norepinephrine fibers suggests that modifications in the serotonin transmission pathway contribute to the antiemetic effect. The neurochemicals released through acupoint stimulation desensitize the vomiting center in the brain, thus preventing nausea and vomiting (10, 31, 68), and #4 The perioperative anesthetic dose, recovery, complications, and prognosis (34-37)are critical areas of study. Additionally, topics such as acupuncture analgesia (27)and reproductive medicine (41) are also included. Finally, we also found that the combination of acupuncture and NIN techniques is in its infancy for the emerging topic area of “mental health,” such as depression (69), Postoperative Cognitive Dysfunction (70), post-stroke depression (71). These findings suggest that future research hotspots in this field will increasingly concentrate on subjects such as analgesia, postoperative complications and mental health.

### 3.3 Strengths and limitations

This study systematically reviews the publication trends and research hotspots in the field of acupuncture and NIN technology integration over the past two decades. It reveals the knowledge structure of this field, assesses the influence of research in this area, and identifies future research trends. In addition, this study uses different software to visually analyze relevant literature in the field of acupuncture and NIN technology integration and provides a comprehensive overview of the development of this field over the past 20 years. It summarizes the current state and cutting-edge research in this field, providing researchers with direction and ideas.

However, this study also has several limitations. First, we only used the WOSCC database for analysis. Although this database contains high-quality academic papers with global influence, the single source of data may limit the breadth of the conclusions of this study. Second, the involvement of three researchers in the article screening process may have introduced subjective factors, resulting in a high degree of subjectivity in the research conclusions and thereby affecting the objectivity of the study. Third, although we retrieved 803 relevant literature articles on acupuncture combined with NIN technology over the past two decades, our understanding of this field remains superficial, and the study cannot fully replace qualitative analysis. Additionally, this study did not stratify the included research publications based on quality and did not assess the methodological quality or clinical impact of the included studies. This may reduce the reliability of the research results and increase the misleading nature of the conclusions. Nevertheless, bibliometrics has had a profound impact on academic research, academic evaluation, and academic dissemination. In the future, with the development of big data technology, artificial intelligence technology, and the advancement of multidisciplinary integration, bibliometrics will have broader application prospects.

## 4 Conclusion

This bibliometric analysis examines the overall trends in publishing as well as the changing patterns of publications indexed in the Web of Science database over the last two decades. It highlights possible collaborators, institutions, research hotspots, keywords, countries, co-cited works, and emerging research trends in this area, which will assist in steering new research paths toward integrating acupuncture with NIN technology. Exploring the potential mechanisms that underpin the synergy between acupuncture and NIN technology, alongside the active employment of various innovative methods and concepts, will signify a crucial developmental trajectory for enhancing clinical treatment strategies in the future.

## Data Availability

The raw data supporting the conclusions of this article will be made available by the authors, without undue reservation.

## Abbreviations

NIN: Non-invasive neuromodulation
TDCS: Transcranial direct current stimulation
tACS: Transcranial alternating current stimulation
TMS: Transcranial magnetic stimulation
TAS: Transcutaneous acupoint stimulation
TUS: Transcranial ultrasound stimulation
EOPs: Endogenous opioid peptides
CSF: Cerebro-Spinal Fluid
SP: Substance P
CGRP: Calcitonin gene-related peptide. Peptide
WOSCC: Web of Science Core Collection
LLR: The log-likelihood ratio
SCI: The Science Citation Index
JCR: Journal Citation Reports
TFP: Three-Field Plot
TEAS: Transcutaneous electrical acupoint stimulation
PONV: Postoperative nausea and vomiting
PON: Postoperative pain
EAE: emergence agitation
PGD: Postoperative gastrointestinal dysfunction
NSAID: Nonsteroidal anti-inflammatory drugs
EA: Electroacupuncture
PND: Perioperative neurocognitive disorders
CNS: Central nervous system
TI: Temporal Interference
ARC: The arcuate nucleus of the hypothalamus
PAG: Periaqueductal gray matter
POMC: Proopiomelanocortin
