# Supplementary material for: Hotspots and trends in acupuncture combined with non-invasive neuromodulation technology in the past 20 years: a bibliometric analysis
Source: Front Neurol. 2025 Sep 9;16:1511655. doi: 10.3389/fneur.2025.1511655 (PMC12456029; doi:10.3389/fneur.2025.1511655)
Supplement: Supplementary file 1 [file Data_Sheet_1.docx]

| Supplementary Appendix 1: Search strategy for the Web of Science Core Collection (WoSCC). | | |
| --- | --- | --- |
| # | Search Query | Results |
| 1 | ((((((((((TS=(Magnetic Stimulations, Transcranial)) OR TS=(Magnetic Stimulation, Transcranial)) OR TS=(Stimulations, Transcranial Magnetic)) OR TS=(Stimulation, Transcranial Magnetic)) OR TS=(Transcranial Magnetic Stimulations)) OR TS=(Transcranial Magnetic Stimulation, Paired Pulse)) OR TS=(Transcranial Magnetic Stimulation, Repetitive)) OR TS=(Transcranial Magnetic Stimulation, Single Pulse)) OR TS=(TMS)) OR TS=(repetitive transcranial magnetic stimulation)) OR TS=(rTMS) | 42109 |
| 2 | ((((((((((((((((((((((((TS=(transcranial direct current stimulation)) OR TS=(tDCS)) OR TS=(Anodal Stimulation Transcranial Direct Current Stimulation)) OR TS=(Anodal Stimulation tDCS)) OR TS=(Anodal Stimulation tDCSs)) OR TS=(Stimulation tDCS, Anodal)) OR TS=(Stimulation tDCSs, Anodal)) OR TS=(tDCS, Anodal Stimulation)) OR TS=(tDCSs, Anodal Stimulation)) OR TS=(Cathodal Stimulation Transcranial Direct Current Stimulation)) OR TS=(Cathodal Stimulation tDCS)) OR TS=(Cathodal Stimulation tDCSs)) OR TS=(Stimulation tDCS, Cathodal)) OR TS=(Stimulation tDCSs, Cathodal)) OR TS=(tDCS, Cathodal Stimulation)) OR TS=(tDCSs, Cathodal Stimulation)) OR TS=(Transcranial Alternating Current Stimulation)) OR TS=(Transcranial Random Noise Stimulation)) OR TS=(Repetitive Transcranial Electrical Stimulation)) OR TS=(Transcranial Electrical Stimulation)) OR TS=(Electrical Stimulations, Transcranial)) OR TS=(Electrical Stimulation, Transcranial)) OR TS=(Stimulations, Transcranial Electrical)) OR TS=(Stimulation, Transcranial Electrical)) OR TS=(Transcranial Electrical Stimulations) | 14150 |
| 3 | (((((((((((((((((((((((((((((((((TS=(Transcranial electric stimulation)) OR TS=(Transcutaneous Nerve Stimulation)) OR TS=(Nerve Stimulation, Transcutaneous)) OR TS=(Stimulation, Transcutaneous Nerve)) OR TS=(Electric Stimulation, Transcutaneous)) OR TS=(Stimulation, Transcutaneous Electric)) OR TS=(Transcutaneous Electric Stimulation)) OR TS=(Percutaneous Electric Nerve Stimulation)) OR TS=(TENS)) OR TS=(Transdermal Electrostimulation)) OR TS=(Electrostimulation, Transdermal)) OR TS=(Percutaneous Electrical Nerve Stimulation)) OR TS=(Transcutaneous Electrical Nerve Stimulation)) OR TS=(Electrical Stimulation, Transcutaneous)) OR TS=(Transcutaneous Electrical Stimulation)) OR TS=(Analgesic Cutaneous Electrostimulation)) OR TS=(Cutaneous Electrostimulation, Analgesic)) OR TS=(Electrostimulation, Analgesic Cutaneous)) OR TS=(Electroanalgesia)) OR TS=(Electroanalgesias)) OR TS=(Percutaneous Neuromodulation Therapy)) OR TS=(Neuromodulation Therapy, Percutaneous)) OR TS=(Percutaneous Neuromodulation Therapies)) OR TS=(Therapy, Percutaneous Neuromodulation)) OR TS=(Percutaneous Electrical Neuromodulation)) OR TS=(Electrical Neuromodulation, Percutaneous)) OR TS=(Electrical Neuromodulations, Percutaneous)) OR TS=(Neuromodulation, Percutaneous Electrical)) OR TS=(Neuromodulations, Percutaneous Electrical)) OR TS=(Percutaneous Electrical Neuromodulations)) OR TS=(transcranial alternating current stimulation)) OR TS=(tACS)) OR TS=(Transcranial ultrasound stimulation)) OR TS=(TUS) | 69268 |
| 4 | ((((TS=(Optogenetic)) OR TS=(Optogenetic Techniques)) OR TS=(Optogenetic Technique)) OR TS=(Technique, Optogenetic)) OR TS=(Techniques, Optogenetic) | 8707 |
| 5 | #4 OR #3 OR #2 OR #1 | 124611 |
| 6 | (((((((((TS = (acupuncture therapy)) OR TS = (acupuncture)) OR TS = (acupuncture point)) OR TS = (Acupuncture, Ear)) OR TS = (body acupuncture)) OR TS = (Auricular Acupuncture)) OR TS = (Electroacupuncture)) OR TS = (electroacupuncture)) OR TS = (Moxibustion)) OR TS = (scalp acupuncture) | 27542 |
| 7 | #6 AND #5 | 1185 |
| 8 | #6 AND #5 and 2023 or 2022 or 2021 or 2020 or 2019 or 2018 or 2017 or 2016 or 2015 or 2014 or 2013 or 2012 or 2011 or 2010 or 2009 or 2008 or 2004 or 2005 or 2006 or 2007 (Publication Years) | 943 |
